# Tourism, Immigrants and Lifestyle Entrepreneurship: The (In)coming of People as a Key Factor for Sustainability of Low-Density Territories—A Case Study in Portugal

**DOI:** 10.1007/978-3-030-65524-2_7

**Published:** 2021-03-18

**Authors:** Anabela Dinis

**Affiliations:** 1 Higher Institute of Accounting and Administration (ISCA-UA), University of Aveiro, Aveiro, Portugal; 2 School of Technology and Management (ESTGA), University of Aveiro, Águeda, Portugal, Centre for Research in Higher Education Policies (CIPES), Matosinhos, Portugal; 3 Higher School of Technology and Management, Polytechnic of Guarda, Research Unit for Inland Development (UDI-IPG), Guarda, Portugal; 4 School of Technology and Management (ESTGA), University of Aveiro, Águeda, Portugal, Centre for Research in Higher Education Policies (CIPES), Matosinhos, Portugal; Management and Economics Department, University of Beira Interior, Covilhã, Portugal

## Abstract

Using the lens of the new patterns of mobility and lifestyle entrepreneurship in the context of counterurbanization movements, this chapter explores the relationship between tourism and immigration, beyond the traditional approach of immigrants as tourism entrepreneurs. The study focusses on a Portuguese rural county, Penamacor, which, for several decades, has suffered a continuous exodus of population and the consequent aging of the remaining population but where, recently, there was a spontaneous phenomenon of foreign people arriving and settling in the area. Thus, through the case of Penamacor, this study aims to answer the following questions: Who are these migrants, and what are their motivations for mobility and to settle in the territory? Are they all the same? How do they make a living in Penamacor? In particular, it seeks to understand whether entrepreneurship (in tourism or other sectors) is a possibility of income generation for these immigrants. Furthermore, it intends to understand what the impact of these immigrants in the territory is, concerning the creation of wealth and well-being in the community. Do they act as community entrepreneurs? Does their presence in the territory generate other mobility flows, through the attraction of other (family and friends) tourists or immigrants?

## Introduction

In a global economy dominated by large urban centres that concentrate a great part of the population and economic activities, there is a vast geographical extension of rural territories. Although these rural areas are highly diverse, a large part of them are lagging regions characterized by progressive abandonment of the traditional activities linked to the primary sector and continuous exodus which translates into lower population densities and an aging population. In these circumstances the viability of health and education infrastructures, as well as of other services associated with quality of life, are quite low, as well as the prospects of economic development and communities’ sustainability.

Entrepreneurship is often considered as an engine for regional economic development, as it generates growth and serves as a vehicle for innovation and change (e.g. Henderson 2002; Dinis 2006; Huggins and Thompson 2015). However, the promotion of the business phenomenon seems particularly difficult in low-density areas that normally present greater limitations on human, material and financial levels as well as in market demand, when compared to urban areas (Dinis 2006).

In the academic sphere, several studies have sought to examine the factors that promote entrepreneurship in rural and peripheral areas as well as the relationships between the creation of new activities and the local populations (Dinis 2002, 2006; North and Smallbone 2006). Nevertheless, several works have reported that the main actors of rural economy diversification were from outside the local community, associating the establishment of small businesses in rural areas to progressive counterurbanization processes (Gorton et al. 1998; Paniagua 2002; Bosworth 2006; Kalantaridis and Bika 2006; Stockdale 2006; Kalantaridis 2010; Akgün et al. 2011; Herslund 2011; Pallarès-Blanch et al. 2014). This is explained because migrants from urban to rural areas tend to have significant business know-how and the capital to invest in their destination rural areas but also have higher qualifications and larger world perspectives (Marchant and Mottiar 2011; Dinis 2009; Klapper et al. 2018). Specifically, rural tourism is an attractive sector for urban immigrants because it gives them the opportunity of living in the countryside, whilst the cost of setting up the business is relatively low, especially when they can invest the profits from the sale of an urban residence (Hoggart and Buller 1995; Iversen and Jacobsen 2016; Mitchell and Shannon 2018; Möller and Amcoff 2018). However, the relationship between tourism and immigration is not limited to the above, i.e. immigrants as tourism entrepreneurs. Williams and Hall (2000, 2002) highlighted the overlaps between both fields of inquiry and also the symbiotic relationship between them. In 2002, these authors classified the studies that explored the tourism-migration nexus in three main themes: (i) tourism and labour migration (e.g. migrants that are attracted by tourism labour opportunities); (ii) tourism and consumption migration (e.g. tourists that turn into lifestyle migrants); and (iii) visiting friends and relatives tourism—which is an outgrowth of migration but also has the capacity to generate new migration and mobility flows. In spite of this classification, these authors also claim the need to have a holistic approach to the study of tourism-migration relationships.

In the European context, there are some empirical studies exploring the nexus between tourism and migration; however, they are mainly concentrated in northern Europe (e.g. Lundmark 2006; Iversen and Jacobsen 2016; Lundmark et al. 2014; Eimermann 2015; Eimermann et al. 2017) or in touristic places in southern Europe (e.g. Williams et al. 1997; O’Reilly 2003). To date, with some exceptions (e.g. Paniagua 2002; Cunha 2018; Barbosa et al. 2020), there is very little empirical evidence of the participation of urban immigrants in diversification activities and also about the motivations, processes and impacts of settlement of international immigrants in very low density and peripheral rural areas in southern Europe. The relative paucity of studies about migrants in these rural areas may be explained by the fact that research into rural development tends to fail at distinguishing the geographical origin of those involved (Paniagua 2002) but also by the fact that, in several regions, this kind of mobility is a new phenomenon.

In Portugal the most rural and peripheral regions are located in the interior of the country. For several decades these regions have suffered a continuous exodus of population and the consequent aging of the remaining population. Contrary to what happens in other countries, with similar problems (see, for instance, the case of Sweden as described by Cassel (2008), Eimermann (2015) and Eimermann et al. (2017)) no rural place marketing efforts have been made, and other political actions have not been able to reverse this situation. Despite this, recently, there has been a spontaneous phenomenon of foreign population coming to some of these territories. This phenomenon has already started to attract the attention of the media and some researchers, but still very little is known about who these immigrants are, why they migrate and what the impact of such mobilities on local communities is.

The Portuguese municipality of Penamacor, being one of the less densely populated municipalities and, according to the last census (INE 2011), the most aged in Portugal, is a paradigmatic case of this phenomenon. In 2012, one of the most widely circulated newspapers in the country reported “the death” of some villages in this municipality (*Público,* 16 July 2012). However, in recent years, there has been a very significant increase in foreign immigrants. Today, it is the municipality that has the highest rate of foreign residents in the interior: almost 10% of the population. A previous empirical study (Cunha 2018) showed that these incomers have lifestyle motivations. However, they are treated as a homogenous group, and little is said about their mobility patterns or their impact in the territory, namely, through their entrepreneurial activities. Also, as highlighted by Cunha (2018: 75), “another dimension of the phenomenon to be explored is to assess the impacts that inevitably the establishment of these expats have on local communities in all areas”.

Thus, through the lens of the new patterns of mobility and lifestyle entrepreneurship, the case of Penamacor is analysed seeking to further explore the following questions: Who are these migrants, and what are their motivations for mobility and to settling in the territory? Are they all the same? How do they differ concerning their lifestyle motivations, their demographic characteristics, their occupations and their patterns of mobilities and spatial frame of action? How do they make a living in Penamacor? In particular, it seeks to understand whether entrepreneurship (in tourism or other sectors) is a possibility of income generation for these immigrants. Furthermore, it intends to understand what the impact of these immigrants in the territory is, namely, in the creation of wealth and well-being in the community. Do they act as community entrepreneurs? Does their presence in the territory generate other mobility flows, through the attraction of other (family and friends) tourists or immigrants?

This case study (Stake 1995; Yin 2009) has an exploratory nature and applies an abductive methodology (Dubois and Gadde 2002) based in a literature review, primary data (an interview with a key informant) and secondary data (TV and other media documentaries, written reports and statistics)—see Appendix for more details. Qualitative data, from primary (personal interview) and secondary sources of information (interviews to Penamacor’s immigrants in the media and in the work of Cunha 2018), were analysed through content analysis: first reviewing the interviews and all the media information and then, based in the research questions, selecting (and transcribing) the relevant statements, following a process of condensation, coding and categorization that allowed to define the themes (underlying meanings) presented in the paper.

After this introduction, the chapter continues with a literature review about the new mobilities, besides tourism and traditional immigrants. It follows with a discussion about lifestyle entrepreneurship in rural areas and its relation with tourism, travellers and migrants. In Sect. 4, Penamacor’s case is presented and discussed in light of the questions raised. Finally, some concluding remarks are made under a development perspective.

## New Mobilities: Between Tourism and Migration

In 2000, Allan Williams and Michael Hall, argued that there was a weak conceptualization of the differentiation between migration and tourism, which contributed to neglecting the relationship between both. In their work they distinguish and discuss both concepts.

Migration is usually defined spatially as a movement from one territory to another that implies some permanence or an intention of settling permanently or temporarily. Tourism, in turn, is generally defined by three main features: (i) it occurs outside the normal place of residence, (ii) it is of a temporary short-term character, and (iii) destinations are visited for purposes other than taking up permanent residence or remunerated employment (Williams and Hall 2000). Thus, being both tourism and migration forms of mobility, *tourism* is traditionally associated to non-permanent mobility to other areas, usually by motives of leisure (or non-remunerated work) and associated to pleasure, adventure, discovery or other “positive” sensations/experiences, whilst traditional *migration* is related with a mobility for long permanence/settling, usually by motives of work, i.e. associated with lack of work opportunities in the region/country of origin.

However, demographic, social, economic, technological and political changes^1^ resulted in an increase of mobility lifestyles in all ages, young adults and seniors and in mobilities mixing different rationalities, as economic with recreational or even ideological or humanitarian motives. This is the case of lifestyle migrants who choose to pursue a new life in the new locale by motives that often involves a new work-life balance, a better quality of life and freedom from prior constraints (Benson and O’Reilly 2009, 2016; O’Reilly and Benson 2009; Ibrahim and Tremblay 2017; Sun and Xu 2019). Lifestyle migration has been increasing in recent years and has been grounded on changes in value systems and facilitated by teleworking (Williams and Hall 2000). Benson and O’Reilly (2016) refer to lifestyle migration as a conceptual framework that focusses specifically on the motivations behind migrations, broadly described as the search for a better way of life whilst also considering it “a process rather than a one-off act completed upon arrival at the destination” (p. 21). In this sense and according to Hoey (2005), for lifestyle migrants, the choice made of *where* to live is, in fact, also one about *how* to live. But lifestyle migrants are not a homogeneous category. Benson and O’Reilly (2009) define lifestyle migration as the migration of “relatively affluent individuals, moving either part-time or full-time, permanently or temporarily, to places which, for various reasons, signify for the migrants something loosely defined as quality of life” (p. 621). Thus, concerning the permanence, lifestyle migrants include those that decided to stay permanently in the chosen location and those who, as “nomads,” lead a peripatetic lifestyle shifting between two or more homes—a form of mobility that Williams and Hall (2002: 7) denominated “circulation” as opposed to migration.

**Fig. 1:**
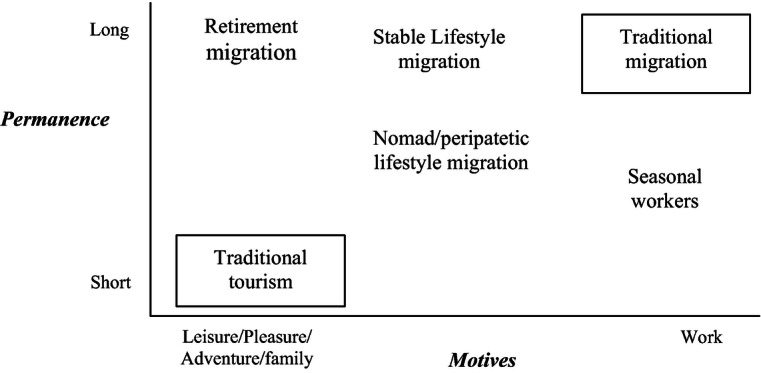
Forms of mobility and the evolving tourism-migration nexus. Source: Elaborated by the author

Summing up, new forms of mobility have emerged, besides traditional tourism and traditional migration, located along a two-dimensional *continuum* that shaped the evolving tourism-migration nexus, as shown in Fig. 1.

The nexus between tourism and migration is not restricted to the areas of overlapping described above. It also exists in the form of symbiotic relationships, as noted by Williams and Hall (2000). In fact, many forms of migration generate tourism flows through the geographical extension of friendship and kinship networks. These flows are, often, bidirectional, i.e. friends and family travel to visit the new place of migrants’ residence; migrants, in turn, become tourists in returning to visit friends and relatives in their areas of origin. The inverse may also happen, i.e. tourism generate migration. This is the case of touristic places that generate labour demand that is not met locally, stimulating labour migration (Lundmark 2006). In addition, tourism may contribute/help to find the right place to settle as immigrant, whether for motives of work, lifestyle or retirement. These links, not being new in the history of mankind in the current era of a globalized world and increasingly mobile societies, are not only becoming more important and relevant but also more and more expressed on the international scale.

Williams and Hall (2002) presented a typology of five main forms of migration related with tourism (see Table 1). The main purpose of this typology is to differentiate whether the migrant aims to work in the tourism industry (production-led migration) or to enjoy the same environment as tourism (consumption-led migration).

**Table 1:** Tourism-related migration: selected characteristics

	Mobility		Age		Property Ownership
	Temporary	Permanent	Younger	Older	
*Production-led migration*					
Labour	x	x	x		
Entrepreneurial		x	x		x
Return		x	x	x	x
*Consumption-led migration*					
Economically active		x	x		x
Retirement	x	x		x	x

Source: Williams and Hall (2002:26)

The typology recognizes the existence of both temporary mobility and permanent migration. It also distinguishes the several types of migration along two other dimensions: migrants’ age and property ownership in the destination. It includes three forms of *production-led migration*, differentiated by the direction of the flow (emigration or return) and position in the economic structure (worker or entrepreneur), and two forms of *consumption-led migration,* differentiated in relation to whether individuals are still economically active or retired. Their links with tourism are in many ways similar to those for retirement migrants: search of spaces for tourism/life experiences and amenity seeking. Both cases of consumption-led migration can be related with lifestyle migration.

In spite of the important advance in the discussion of the tourism-migration nexus, Williams and Hall’s typology (Williams and Hall 2002) does not explicitly consider the possibility of consumption-led migrants (e.g. lifestyle migrants) being also entrepreneurs. This blurring of the borders between production-led migration and consumption-led migration was already studied by Williams et al. (1989) and has also received attention from other authors, as will be discussed in the next section.

## Tourism, Travellers, Migrants and Lifestyle Entrepreneurship in Rural Areas

### Lifestyle Entrepreneurship: Around Definitions and Typologies

Many researchers and policymakers have noted and encouraged entrepreneurial ventures as a mean of economic growth (Acs 2006; Acs et al. 2018; Baumol and Strom 2007; Braunerhjelm et al. 2010; Schumpeter 1934; Wennekers and Thurik 1999). However, in recent years, an increasing number of research studies analysed the phenomena of lifestyle entrepreneurs as the opposite of the growth-oriented or typical Schumpeterian entrepreneurs (Ateljevic and Doorne 2000; Peters et al. 2009; Cederholm and Hultman 2010; Claire 2012; Bredvold and Skålén 2016; Cunha et al. 2018; Sun et al. 2020). The contrast between entrepreneurs who develop businesses for profit and those who are motivated by lifestyle has been the focal point of much discussion in research focused on tourism entrepreneurship (e.g. Williams et al. 1989; Shaw and Williams 1998; Getz and Carlson 2000) but also on entrepreneurship in rural areas (e.g. Henderson 2002; Dinis 2009) as well as on migrant entrepreneurship (e.g. Bosworth 2006; Hedfeldt and Lundmark 2015).

Lifestyle entrepreneurs have been defined as a specific type of entrepreneurs whose aim is finding a sufficient and comfortable way of living and less focussed on profit and growth. In other words, they are primarily motivated by the need to succeed at living a certain quality of life by maintaining an income which allows them to survive (Marcketti et al. 2006; Marchant and Mottiar 2011; Bredvold and Skålén 2016). This quality of life could be defined by the possibility to maintain or practice a hobby and/or to balance work, family and social needs. In some cases, as those described by Ateljevic and Doorne (2000), lifestyle entrepreneurs consciously reject economic and business growth opportunities as an expression of their sociopolitical ideology, which “do not subscribe to the inevitable path of ‘progress’ as an end in itself” (p. 381). In these cases, the “market ethos” (p. 379) is rejected in favour of personal values, beliefs, interests and passions, usually related with sustainability and connection with nature, as motivating factors for doing business.

But, as also argued by Bredvold and Skålén (2016), this kind of dichotomy between commercial and lifestyle entrepreneurship is a simplification, because it is possible to find “hard-core” lifestyle entrepreneurs, entrepreneurs motivated by economic factors and entrepreneurs motivated by both lifestyle and economic factors (e.g. Shaw and Williams 1998, 2004).

One of the most well-known typologies of lifestyle entrepreneurs that make evident this diversity is that of Shaw and Williams (1998) that distinguishes between “non-entrepreneurship” and “constrained entrepreneurs”. The first group shows similarities with the “pure” lifestyle entrepreneurship, as they have moved into tourism destinations for non-economic reasons; they have established enterprises (mainly with personal savings) and enjoy being their own boss. Many of these non-entrepreneurs are owners who have retired from former professions and perceive tourism and hospitality small businesses as a way to enjoy a nice destination whilst generating some income to sustain their lifestyle. This group is often characterized by ageing owners or retirees and were labelled “non-entrepreneurs” because they showed a lack of business experience and “entrepreneurial activity was extremely limited” (Shaw and Williams 2004, p. 102). The second group of “constrained entrepreneurs” constitute younger people with economic growth motives, but they demonstrate many lifestyle motives to explain their activities. The capital required is family raised and have former professional experience in tourism and other industries. They also demonstrate some entrepreneurial attitudes towards innovation and product development, as well as towards customer values and needs. They are “constrained” by their desire for a certain lifestyle as well as the business, but, contrary to non-entrepreneurs, they are willing to grow the business given the right training and support.

Another typology distinguishing entrepreneurs with lifestyle motivations is the typology presented by the author in a previous study. In that study, Dinis (2009) identified two types of entrepreneurs in rural areas. One type is the “owner-entrepreneurs”, usually native people, whose main motivation is providing a family income. They do not have the desire to leave the rural place, neither the desire (or capability) to build high-growth business. In these cases, the creation of self-employment and the generation of an income source (exclusive or supplementary) are seen as the success criterion. This kind of entrepreneurship also often allows to preserve or valorize local/endogenous resources (e.g. natural resources, traditional arts, other aspects of local culture, etc.). The benefits of the existence of these kind of entrepreneurs relates with the quality of life in local communities, providing many of the services needed by local residents but also, given “personality and charm”, to local economy that attracts many people to shop and live in rural communities (Henderson 2002:49). The other type of entrepreneurs, called “entrepreneurial-entrepreneurs”, are those who enjoy and choose to live in a rural place but they are more innovative, more aware of the rural resources in the global market and develop a strategic vision of the business. They also differ from the previous mainly for their “expanded life experience”. Many of them have lived experiences outside the local scope, such as studying or living in other places (in a city or abroad), traveling frequently and/or interacting with a larger social network (reaching individuals in urban/international contexts and/or in central positions). Besides that, they are usually younger and with a higher level of education (not necessarily higher education) than the “owner-entrepreneurs” type.

### The Impact of Lifestyle Entrepreneurship in Rural Areas: A Discussion Around Tourism, Travellers and Migrants

Adopting the economic theory perspective, Peters et al. (2009) define lifestyle entrepreneurs as those entrepreneurs that accept suboptimal levels of production. These authors, based in a literature review, also point several characteristics of lifestyle entrepreneurs such as limited capital, lack of skills, irrational management, low innovation, and unwillingness to cooperate. This definition helps to understand why some authors claim that this type of entrepreneurs constrains regional economies and create problems for firm survival (e.g. Williams et al. 1989; Getz and Carlson 2000; Peters et al. 2009). However, others maintain that despite the fact that lifestyle entrepreneurs do not follow economic motives, their contribution to economic welfare and customer satisfaction should not be underestimated (e.g. Ateljevic and Doorne 2000; Dinis 2009; Marchant and Mottiar 2011). As noted by Peters et al. (2009) lifestyle entrepreneurs often get involved in business because they are experienced consumers, who either make a profession out of their hobby or seek optimal customer oriented solutions still not provided by the market. Thus, many lifestyle entrepreneurs can be seen as lead users who can be important sources of product/service innovations. The business creation can happen by “coincidence”, i.e. a situation “evolving organically with one incident leading to another” (Cederholm and Hultman 2010: 21) or meant to make that settling choice economically viable. Furthermore, as concluded by Ateljevic and Doorne (2000), even in the cases of “ideological” lifestyle entrepreneurs, the conscious rejection of a profit-driven orientation does not necessarily result in financial suicide or developmental stagnation but rather provides opportunities to engage with “niche” market consumers who share the same values of the lifestyle entrepreneur. These consumers, with values embracing environmental and sociocultural integrity, identify themselves as “travellers”, as a distinct identity from the more traditional “tourist” (the “others”) whose activities are pre-planned and packaged by the industry, who seek hedonistic and frivolous experiences. These authors also concluded that the lifestyle entrepreneurs of their study were immigrant*.* As they state:

These ‘outsiders’ are often individuals who previously visited the area as ‘independent travellers’, yet in making this move seek an opportunity to engage in extended lifestyle experiences, which reflect the traditional motivations of the ‘backpacker’. (p. 386)

The relevance of past experience of travel is also highlighted by Marchant and Mottiar (2011). In some cases, the travel facilitated experiences that influence individuals’ decisions about what they wanted to (or can) do with their lives. In other cases, experiences and knowledge from one place made individuals aware of the potential for offering the same service or product in other places. So, in many ways, travel has been a catalyst for their entrepreneurial journey. Thus, it was no coincidence that many of the lifestyle entrepreneurs were “travellers” themselves, motivated by their experiences and/or values often inscribed in the sustainability paradigm.

In the “ideological” cases, paradoxically, the search to escape from a “suffocating” market environment has provided a niche opportunity to engage with that market on their own terms and to sustain their businesses. Furthermore, despite their efforts to limit the growth of their own businesses, the innovative and creative attributes of these individuals make them dynamic elements in the economy, as those entrepreneurs described by Schumpeter (1942). This paradox illustrates the extent to which consumption and production are intertwined and provides research opportunities to disentangle the conventional polarization of entrepreneurship in terms of production and consumption.

Besides that, often lifestyle immigrant entrepreneurs have levels of education higher than native people, and their life experiences make them good communicators, enjoying interacting with people (Marchant and Mottiar 2011). Also, they have relied at the start-up phase on imported financial capital, albeit largely their own. They have also brought with them internalized skills, ways of thinking, priorities and preferences (Klapper et al. 2018). These attributes can provide potential benefits for the local destination areas and community. Some of them are very much involved in the local community, coming together to run local initiatives and being actively involved, for instance, in local chambers of commerce or business organizations. Thus, in addition to personal satisfaction, lifestyle entrepreneurship can also enhance well-being at the community level (Ateljevic and Doorne 2000; Marcketti et al. 2006), making them, also, communitarian entrepreneurs (Cornwall 1998; Johannisson and Nilsson 1989), i.e. important parts of community building and a powerful element in processes of social change.

But the relationship with community has a symbiotic nature. In fact, as pointed by Klapper et al. (2018) in a context of tight resource constraints, i.e. of limited availability of financial capital, accumulation of social capital (Bourdieu 1980; Putnam 1993) is fundamental to the survival and prospering of the businesses. That is, lifestyle entrepreneurs are able to survive commercially, partly, because their financial expectations are relatively low and, partly, because they can rely on each other and are able to deploy a range of coping practices. As noticed by Ateljevic and Doorne (2000, 386) “the growth of many of these businesses (…) has been via the facilitation of community, family and friendship relationships based on a common set of values”.

## The Case of Penamacor

### Characterization of the Territory

**Fig. 2:**
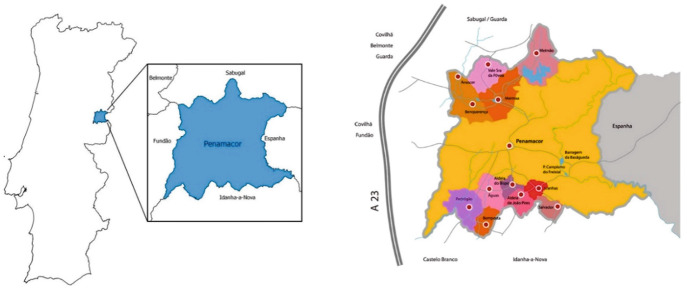
Municipality of Penamacor: classification in the national territory and main localities. Source: Vilas Boas et al. (2015) and http://www.cm-penamacor.pt/cmp/index.php/conhecer/caraterizacao

The municipality of Penamacor is located in Centro region (NUTS II), in Beira Baixa (NUTS III). It is limited, in the east, by the Spanish Extremadura region. The municipality included 12 villages: Águas, Aldeia do Bispo, Aldeia de João Pires, Bemposta, Pedrógão, Aranhas, Benquerença, Meimoa, Meimão, Penamacor, Salvador and Vale da Senhora da Póvoa (see Fig. 2). All together, they possess a population of less than 5000 inhabitants, making Penamacor one of the municipalities with the lowest population density in Portugal. It is also one of the Portuguese municipalities with the highest aging rate (see Table 2). For this reality contributed a strong and continuous emigration flow of the local population, since the end of the 1950s of the last century, with special incidence in the decades from the 1960s and 1970s, essentially targeting Europe, in a first phase, and then the national coast, mainly Lisbon, in search of better living conditions. In 2012, one of the most popular newspapers (*Público*, edition of July 16), in a report about this municipality, announced the death of its small villages, declaring them “in extinction”.

**Table 2:** Resident population, population density and aging index in Portugal, Centro Region, Beira Baixa and Penamacor municipality (estimates at December 31, 2018)

Territory		Resident population Individual	Population density^a^	Aging index^b^ Ratio—%
NUTS I	Portugal	10,276,617	111,5	157,4
NUTS II	Centro	2,216,569	78,9	196,6
NUTS III	Beira Baixa	80,782	17,6	281,9
County	Penamacor	4831	8,6	630,4

^a^Average number of individuals per km^2^

^b^The relationship between the number of elderly people and the young population in a certain region. More specifically, it is the ratio of the number of elderly persons aged 65 and over (when they are generally economically inactive) to the number of young persons (from 0 to 14).

Source: PORDATA, Data Sources: INE—Annual Estimates of the Resident Population; Data Sources: IGP—National Cartographic Series at 1:50,000 scale and Official Administrative Letter of Portugal—CAOP 2009.0. Last updated: 2020-02-07

In view of this demographic scenario, economic activity naturally suffers, conditioned by the lack of entrepreneurship and qualified labour and by the weak expression of the local market. Trade is unable to compete with the nearest urban centres (Fundão, Covilhã and Castelo Branco^2^), and the most relevant productive sector is usually related to local resources, with particular emphasis on forestry and the small agrifood industry based in the olive grove and in the breeding of cattle, sheep and goats. However, agriculture tends to assume subsistence characteristics in the region, due not only to the demographic characteristic of the local population but also to the land tenure characteristics of the farms, which are very parcelled and often abandoned.

### The Immigration Phenomena

Despite this reality, since 2017 the municipality of Penamacor made headlines again, first in the local press, and, soon after, also in the national press and on television. But this time to deal with a striking phenomenon alluded in the headings of the media: “Foreigners are giving life to Penamacor” (*Reconquista*, 23 March 2017), “Penamacor has the highest rate of foreign residents in the interior” *(TVI24*, 2 October 2018), and “The fugitives Brexit are invading the oldest municipality in Portugal” (*Diário de Notícias*, 14 October 2018). In this news, the subtitle advances:

Penamacor was the oldest municipality in the country. Today, it has the highest rate of foreign residents in the interior—almost 10% of the population. They are mainly English, working age and fleeing Brexit. They are buying abandoned farms, they opened an international school, they work online for the whole world. There is a new world in Beira Interior.

**Fig. 3:**
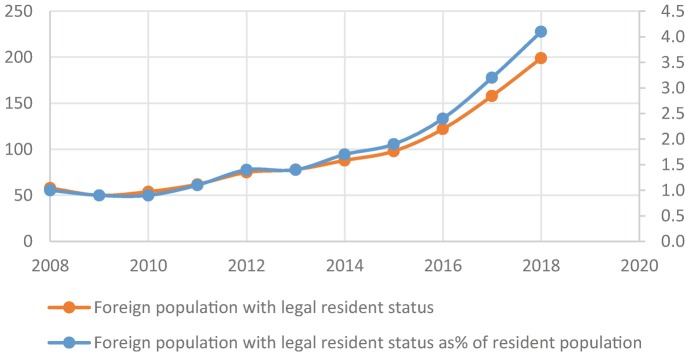
Evolution of foreign people in Penamacor county (2008–2018). Source: Elaborated by the author based in INE, Portugal—Resident Population Annual Estimates | SEF/MAI—Foreign Population with Legal Resident Status. Data provided by PORDATA (last update 2020-02-07)

In 2019, this phenomenon was the subject of a large television report on one of the most popular news channels in Portugal, entitled “Good Morning Penamacor”^3^ (*SIC Notícias*, 27th March), showing and giving a voice to the community of foreigners living in the villages of Penamacor. This situation is also highlighted in the official statistics. Data show that between 2008 and 2018, and especially since 2016, year of the *Brexit* referendum, the percentage of foreign residents in Penamacor increased from 1 to 4.1, corresponding to a 243% increase in the foreign population in this territory (Fig. 3).

And if the accentuated presence of foreigners in interior and less densely populated territories in Portugal is not so usual (the vast majority is located in urban or coastal centres), what makes the case of Penamacor particularly impressive is the strident growth in the last 4 years and the origin of these new inhabitants. In fact, this sharp increase is mainly due to the income of immigrants from the United Kingdom that, in 2018, already represented the majority of the foreign population in the municipality (56. 6%), a much higher weight of British than that registered in any other municipality in Beira Baixa (13.8%), in Algarve (18.3%)—the traditional destination of British people in Portugal—and even more when compared to the average weight of these emigrants in the Centro Region (7%) and in Portugal (5.5%) (Pordata; 2020/INE| SEF/MAI).^4^

**Fig. 4:**
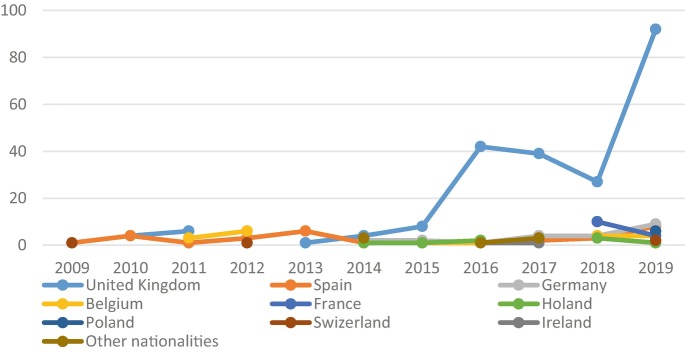
Evolution of registration certificates for foreign residents issued by SEF for the municipality of Penamacor (2009–2019). Elaborated by the author. Source: Serviço de Estrangeiros e Fronteiras (SEF)/Foreigners and Borders Service

Data provided by the municipality of Penamacor, collected from the Foreigners and Borders Service (SEF), provide more accurate and updated information (see Fig. 3). In the period considered, 359 foreign immigrants were registered in the municipality of Penamacor, the majority (65.5%) being immigrants from the United Kingdom.^5^ Figure 4 shows the weight of the different nationalities of foreign immigrants. It should be noted that around 90% of foreign immigrants are from northern and central Europe, with no entries from other continents (e.g. Brazil, Cabo Verde and China) revealing a *sui generis* pattern of immigration in this county.

### Profiles of Foreign Immigrants in Penamacor: Searching for a Typology

Besides the national profile of the foreign immigrants in Penamacor presented in the previous section, it is now intended to further understand who they are and what reasons stand behind their decisions. Cunha et al. (2018), in his work carried out between 2017 and 2018^6^, concluded that the reasons of these new inhabitants for moving to the countryside are related to lifestyle, clearly integrating with the so-called counterurbanization (Gorton et al. 1998). Jammie Malloy (identified as #9 in Appendix), one of the first movers to Penamacor and that nowadays represents a central figure in this foreign community, described the incomers as:

Young people, young families, retired people, people that have businesses online, people who have houses back in their own country that are renting out or have sold to find a better life or a more tranquil life and peaceful. (SIC Notícias, 25/03/2019)

This statement makes it evident that, in spite of the fact that they have their lifestyle motivations in common, they are not all the same, showing differences in their demographic characteristics, personal motivations and, eventually, occupations and patterns of mobility and spatial frames of action, which will be analysed below.

#### Demographic Characteristics

As showed by Cunha (2018) and as evidenced by the other empirical sources (see Appendix), there are immigrants of all ages in Penamacor. According to Cunha (2018) the most represented age groups are those between 22 and 60 years old^7^ and in most cases, the household is composed not only for the couple but also by several children. These characteristics are in sharp contrast to the characteristics of the local population, as shown in Table 1.

#### Motivations

A closer look at the results obtained by Cunha (2018) shows that lifestyle motivations are not homogenous. In fact, a considerable group (44% in his study) expressed the search for an alternative way of living as the main reason, similar to those “pure lifestyle” immigrants described by Ateljevic and Doorne (2000). The lifestyle choice of this type of immigrants is associated with a sociopolitical ideology that both reject the “market ethos and economic rationalities” (Cederholm and Hultman 2010: 17), i.e. the “capitalist world” and is based on a strong commitment with sustainability and connection with nature. However, other immigrants mention reasons with less ideological factors envolved and eventually more self-centered, related with the individual concern of searching for a better way of living either by living a more peaceful life away from the urban fuss or by the possibility of realizing a personal project (agricultural or other) more in line with the reasons described by Benson and O’Reilly (2009) and Ibrahim and Tremblay (2017). This distinction—between socio-ideological motivations vs better-quality life motivations—is also evident in diverse statements of this new immigrants as the examples shown in Box 1.

##### Box 1 Evidences of distinct motivations of Penamacor lifestyle foreign immigrants

**Table:** 

Socio-ideological motivation	Better quality of life motivation
*“Sweden is a country that, in my opinion, is moving a bit more to the right than I am comfortable with, and that’s one of the reasons why we feel very much at home here in Portugal, because Portugal is a country that is very including”* (former leader of the Swedish ecological party) (# 11, SIC Notícias, 25/03/2019) *“…the ability to make my own choices in line with my own ethics. To be able to practice natural farming without interference from governments which are little more than mouthpieces for corporations which are destroying this world with great success. This is not the kind of ‘success’ that I am looking for. I could not achieve this in ‘Great’ Britain so I came here”. (…) “The ugly face of corporatism and how it has insidiously taken over virtually every aspect of modern life; this is true of nearly every western democracy At least it seemed to me that Portugal with its rich rural tradition would be a good place to pursue my dreams” (#6,* Cunha 2018*: 65–66)* *“this is the beginning of the Research Development Center [for sustainability and alternative cities], right here in Village João Pires, center of Portugal”. (…)* (# 15, SIC Notícias, 25/03/2019) *“[U.K.] it’s a mess. Nobody thought that this would be the results [of the Brexit referendum] and this tipped my hand as to think: ‘O.k. This is it now: I’m gonna stay because it’s becoming hostile back home, I feel…’” (#14, SIC Notícias, 25/03/2019)*	*“…I made the decision to radically change my life. And to be honest, I just felt ready for a new adventure! (…) Following the recession in England, where property prices plummeted, I realised that in order to follow my dreams, I would need to find somewhere cheaper to live’” (…) “I love the countryside here – this year in particular there have been so many spring flowers and so many beautiful birds. I also enjoy seeing snakes, lizards and terrapins in the river” (#5,* Cunha 2018*: 65–66)* *“It’s peaceful, is nice landscape, is not so crowed, not so touristic, it’s green, but not so MUCH… (We lived in Pedrogão Grande before and it’s dangerous because of the fires and here is more calm)” (# 16, SIC Notícias, 25/03/2019)* *“The people are very welcoming and it is a pleasant, simple life ‘tranquilito’”. (#9, TVI24, documentary 01-11-2018)* *“Here you can live a very peaceful, quiet, rural life but you are not isolated” (#7b, Diário de Notícais, 19/10/2018)* *“…now we have our own land, where we can live together as a family”(…)* “*I feel happier here. And I like my children to grow in the freedom of the countryside and in nature…”(#4a,* Cunha 2018*:64, 66)* *“The weather is one of the main reasons, at least why I pick Portugal” (#14, SIC Notícias, 25/03/2019)*

#### Occupations, Mobilities and the Spatial Frames of Action

According to Cunha et al. (2018), in their country of origin, immigrants predominantly had professions linked to intellectual and scientific activities (47%), which includes artists, teachers, health professionals, etc., followed by skilled workers in industry, construction and craftsmen (18%) and intermediate technicians and professions (11%), which reflects relatively high levels of qualification, which tend to be superior than native people.

Unlike what happens in the Algarve region^8^ (Guerreiro 2019), most of this population is in working age and still maintains an occupation. The study by Cunha (2018) found that almost all respondents (92%) are engaged in agricultural activities.^9^ However, these activities are often combined with other professional activities, developed locally (see Box 2) or globally (see Box 3), in this later case, making large use of information technologies.

##### Box 2 Local occupations

*“He is an Australian graduate in Physical Education, who was an international hockey player for many years. (…). Now here he is teaching kids of 11, 12 years old”* (*#8, Diário de Notícias*-14/10/2018)

*“At the Jamie Molloy Hamburgers bar and van, it is the meeting point for the foreign community that lives in Penamacor. In addition to this business, Jamie owns the first real estate agency in town”. (#9 TVI 24-02/11/2018)*

*“I’m a carpenter and a builder and I like working with the stone and wood and I do have other forms of trade work but my specialty is carpentry. There are many beautiful buildings out there that need restoration and there’s a lot of people that came out here to set up a home and lots of them abandon places like this and it takes a lot of work to get it in a beautiful place… Yes. I think there should be enough work on here”. (#18b, SIC Notícias-27/03/2019)*

##### Box 3 International/global occupations

*“From here she continues to* ***write for a Pakistani*** *magazine and also his* books. *This at night because the day is spent looking after the vegetable garden where she has everything she needs”. (# 19 TVI 24-01-11-2018)*

*“A* ***professional truck driver****, he* ***regularly returns to England*** *to work for short periods and obtain some savings that will allow him to return to his farm the rest of the year, always taking advantage of the trip to transport and sell some profitable products such as olive oil that he himself produces with the olives of its 100 olive trees but also with all the olives that its neighbors offer it on the tree for not wanting to harvest it”.* (#3, Cunha 2018: 64)

*“I teach English* ***on-line*** *and I teach people from* ***all over the world****. I tend to teach business professional from Asia, Northern Europe, from Spain, Italy, from Africa and Mexico: I have one student that lives in Congo”* (#5, Diário de Notícias, 19/10/2018)

*“[He is] a computer engineer who does 3D projects* ***for the whole world”*** *(#7 Diário de Notícias*-14/10/2018*). “I use to work in a university and I was a computer programmer leading with virtual reality applications (…) Technology, in my point of view is good. In a lot of ways, it’s an enabler (…) makes the world a smaller place, in a lot of ways, it allows people to work on internet. Make possible to live in a rural place like Penamacor and still* ***access to wider world*** *and make a living” (#7 Diário de Notícias*-19/10/2018)

*“I’m psychologist,* ***I work on-line*** *and* ***I have clients all over the world,*** *and run a company with 15 team members, and so that’s why I can stay everywhere”. (#16, SIC Notícias, 25/03/2019)*

Not only for their international occupations but also because of their mobility experiences (see Box 4), the foreign community in Penamacor tends to be “citizens of the world”. Considering their lifestyle attitude and values, as the connection with nature, the concern with sustainability and the need to move away from urban and (massive) touristic areas, they also fit the notion of “travellers” as defined by Ateljevic and Doorne (2000). This is also the perception of the mayor of the county, António Beites when saying “we can see that they are highly travelled people, with a lot of world and a high cultural level” (#1, Diário de Notícias, 19/10/2018).

##### Box 4 Mobility experiences

*“At about 30 years of age they experienced the extraordinary adventure of crossing the Atlantic Ocean in a 6-meter boat, without motor or radio. Leaving* ***Tenerife*** *in the Canaries, for 33 days they only saw sea and sky until they docked on the island of* ***Saint Martin*** *where they stayed for about 35 years and where they had a small sewing business making upholstery for boats, canvas and candle repairs, and collection systems of sunshade / water for yachts. Their decision to move from a small Caribbean island to a small village in the interior of Portugal, in a remote area, is seen as another adventure in their lives, probably the last, they recognize, but just another adventure.* (*#2*, Cunha 2018: 63) *They meet in south of France. “I’m not certainly a major patriotic person, Like I said,* ***I’ve been more than half of my life out of the country****, I’ve been back to England for four weeks in 40 years”. (#2, SIC Notícias 25/03/2019)*

*“I’ve played football* ***Uzbekistan, England and Australia****. (…) I have a Portuguese wife and I met her in Hong Kong when I was professionally athlete in Hong Kong” (#2, SIC Notícias 25/03/2019)*

*“The fight against the Taliban in Afghanistan and Pakistan forced this 62-year-old journalist into exile, refused to stay in* ***Scotland*** *from where she left in 1983 and 30 years later took refuge in Penamacor (#19, SIC Notícias, 25/03/2019) ‘It was necessary for me to leave from* ***Pakistan*** *and I wasn’t sure where I was going to go. So I was going to go first in* ***Portugal****, then in* ***Spain****, then in* ***Corsega****, and* ***Italy*** *and* ***Turkey,*** *but I came to Portugal and I didn’t go any further’” (#19, TVI24, 01-11-2018)*

*“****I’ve travelled all over the world****,* ***it was my dream****, I worked as a flight attendant, then on a cruise and then I met my partner and he always wanted to have land, take care of the land, have animals and then we had a son and everything changed…” (#18 SIC Notícias 25/03/2019)*

*“I’ve spoke to a lot of my friends who voted to leave because I was shocked they did because they knew that it would hurt me in trying to stay here, because* ***it was beautiful before to travel between [the two countries]*** *and not having any issues, and now who knows”? (#14, SIC Notícias, 25/03/2019)*

Both characteristic of higher qualifications associated with liberal and skilled professional backgrounds and the relevance of past travel experience are consistent with other studies (e.g. Marchant and Mottiar 2011; Dinis 2009; Klapper et al. 2018).

The lifestyle characteristics of these migrants configure what Williams and Hall (2002) called *consumption-led migration*, including both economically active and retired migrants. Concerning the patterns of mobility of the active migrants, it is possible to identify at least two patterns: those that oscillate between Portugal and their country of origin [or other countries where they work], as is the case of Ian (see #3 in Box 2), and those who decided to settle permanently in Penamacor.

The first situation, in a “circulation” mode (Williams and Hall 2000), corresponds to *nomad* or the *peripatetic lifestyle migration*, shifting between two or more homes. This form of mobility was also mentioned by Cunha (2018:13) in their work about Penamacor immigrants^10^:

According to [SEF and Penamacor Municipality] (…), these expats adopt a ‘new nomad’ scheme (…), that is, they remain relatively long periods both in Portugal and in the country of origin, first because their financial situation allows them to have this ‘nomadism’ but also, due to the combination of other factors such as the family connections and commitments they maintain at the origin, or the advantages and benefits attributed to the health system and plan that they already had at the time of acquiring the ‘secondary’ property in Portugal.

The second situation corresponds to those earlier described as *stable lifestyle migration* which might be the majority of the immigrants. In fact, Cunha (2018) found that most immigrants (53% of the respondents) intend to stay and live forever in the territory with only about 1/5 with intention of leaving.

**Fig. 5:**
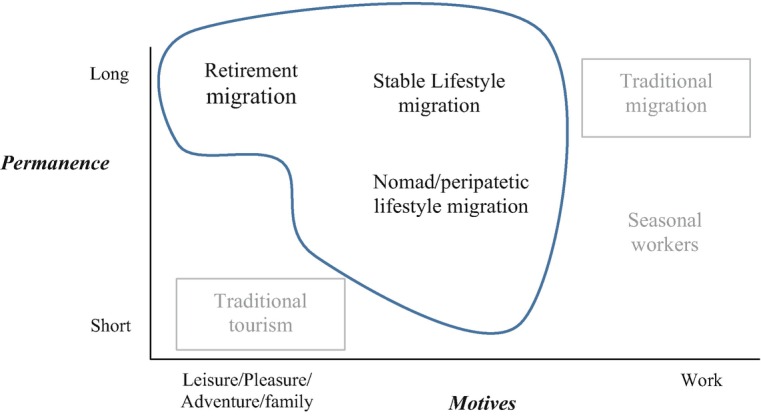
Forms of mobility of Penamacor immigrants. Source: Elaborated by the author

Therefore, in spite of the fact that foreign immigrants in Penamacor have in common their lifestyle motivations and “travellers” characteristic, they are not homogenous in what refers to the motives/concerns and permanence of the stay, fitting in three of the types previously identified: *retired migrants*, *stable migrants* and *nomad migrants* (Fig. 5).

These differences can be better understood in a multidimensional scheme, along four dimensions: (i) age/situation in the labour market (active vs retired); (ii) individual or family lifestyle vs social/ideological motives; (iii) frame of action (occupation); and (iv) duration of permanence in the territory, as follows (Table 3).

**Table 3:** Incomers in rural and low-density areas: selected characteristics

	Retirement migration	Stable lifestyle migration	Nomads
*Age*			
Older/retired	x	x	x
Younger/economically active		x	x
*Concerns*			
Individual/family lifestyle	x	x	x
Social/ideological	x	x	x
*Frame of action/occupation*			
Local		x	
Global		x	x
*Mode of the presence*			
Permanent	x	x	
Circulation	x		x

Source: Elaborated by the author

In spite of their global perspective, these citizens are also closely linked to the local territory, right away, in the physical sense, since the practice of agriculture implies a connection to the land, but also in the sense of community. According to Cunha (2018), a significant number of immigrants (32% of respondents) expressed a desire to be more involved with the local community, probably the group of immigrants who have already taken the decision to settle definitely in Penamacor.^11^

### The Impact of Lifestyle Immigration in Penamacor: Between Accidental/Casual Entrepreneurs and Community Entrepreneurs

As discussed before, entrepreneurship has been considered a central process for economic development. Concerning lifestyle entrepreneurship—here defined as the process of creation of new ventures by people motivated to find a sufficient and comfortable way of living and less focussed on profit and growth—the impact on local economy may be less evident, even though, as shown by several authors (e.g. Paniagua 2002; Dinis 2009; Peters et al. 2009; Pallarès-Blanch et al. 2014), lifestyle immigrants with entrepreneurial ventures can have a significant impact in rural areas.

In Penamacor, the economic impact of the coming of these immigrants was felt immediately in the acquisition of properties that were not occupied. This is evident in the testimonies collected from all sources and also explained by Cunha et al. (2018) in his study:

As a result of the emigration movements registered in the last 4 decades, a significant abandonment of the lands is visible and notorious, with the prospects of reoccupation by their owners being practically null, the overwhelming majority of whom will have remade their lives in the places of destination. Nor is it expected that the second and third generations will reoccupy those same lands, from which they did not derive any economic benefit—on the contrary, if we consider some fiscal obligations—, as well as the nonexistent affective connection that, in many cases, would make return, albeit sporadically, their ancestors. It is mainly these abandoned lands that are now occupied by these new inhabitants, also attracted by their low price. (Cunha 2018: 75)

In fact, this demand for properties created a business opportunity that Jamie Malloy (#9), an immigrant with previous business experience, ended up exploring creating *Quintamaior*, the first State Agency in Penamacor. This started as an informal activity that grew organically and became a firm with two partners in 2018 and generated, in 2019, a new job (see Box 5), a process similar to that described by Cederholm and Hultman (2010). As mentioned by Pedro Agapito^12^ “this could have been created for any people from Penamacor, but it was by them” alluding to their entrepreneurial spirit or “alertness” (Kirzner 1973). This entrepreneurial attitude towards innovation combined with their lifestyle values fits the figure of “constrained entrepreneurs” described by Shaw and Williams (2004) or, considering also their “expanded (global) life experience”, the rural “owner-entrepreneurs” identified by Dinis (2009). This case also illustrates, as stated by several authors, how lifestyle entrepreneurs, being lead users, became promoters of service innovations.

#### Box 5 Jamie Malloy entrepreneurial ventures

*“At the Jamie Molloy Hamburgers bar and van, it is the meeting point for the foreign community that lives in Penamacor. In addition to this business, Jamie owns the first real estate agency in town (…) We [with is partner #10] have the estate agency ‘Quintamacor’ because so many people come in now”. (#9, TVI 24, 01-11-2018)*

*“[He] was one of the first foreigners to emerge in the village. ‘In my other life I was an engineer, responsible for the safety of a gas exploration in Australia. One day I got fed up and opened a bar in Melbourne, but I ended up going bankrupt and going back to Europe. (…) I came here five years ago because I was told that there was really cheap land here. I bought a farm and started to tell some friends, especially English, that there were fantastic opportunities here”. (#9, Diário de Notícias-14/10/2018)*

*“Three years ago he sold the farm to an English couple, bought another. And that was when he created a website to sell properties. ‘In the meantime I decided to make things official and create an agency. Last week I made the sale number one hundred. All farms, and everything to foreigners.’ (…) The Irishman also has a bar, the JCJ, which serves as a meeting point for the foreign community. It serves beef, chicken or vegetarian burgers and fills up in the late afternoon like an English pub. ‘Now I’ve just bought a store and I’m going to open a laundry. Most foreigners live on farms that run on solar energy and can’t get enough energy for a washing machine’”. (#9, SIC Notícias, 25/03/2019)*

*“I’m starting to work to Quintamacor, at the moment, with the guys (…)”. (#17, SIC Notícias, 25/03/2019)*

The impact on the local economy is also extending to small service providers in construction and in agriculture, as testimonies in Box 6 make evident, and, based on Cunha (2018), this impact tends to grow. In fact, this author found that in the near future, a significant number of immigrants (50% of respondents) expected to start an entrepreneurial activity.

#### Box 6 Other entrepreneurial ventures by immigrants

*“I’m a carpenter and a builder and I like working with the stone and wood and I do have other forms of trade work but my specialty is carpentry. There are many beautiful buildings out there that need restoration and there’s a lot of people that came out here to set up a home and lots of them abandon places like this and it takes a lot of work to get it in a beautiful place… Yes. I think there should be enough work on here”. (#18b, SIC Notícias, 25/03/2019)*

*“There is not enough work for me around here as an electrician because there are already local electricians and I don’t want to take any work away from the guys who already established here themselves. I’m trying to get away from that and sort up and to make some progresses with the farming”. (#20, SIC Notícias, 25/03/2019)*

As described by several authors (Ateljevic and Doorne 2000; Marcketti et al. 2006; Kapler 2018), these immigrants have a set of skills and abilities that translate into a benefit for the local community, translating in some cases in new services or innovative activities, as the cases presented above, but also resulting in the promotion of other communitarian initiatives such as the creation of a school with an innovative curriculum (the international school) (see Box 7) or the carrying out of cultural activities such as the collaboration in Penamacor intercultural fair. Being actively involved in local initiatives and “actively seeking closer relationships and initiate inclusive community relationships which emphasise social worth” as described by Ateljevic and Doorne (2000, 386), they, in fact, also act as communitarian entrepreneurs (Cornwall 1998; Johannisson and Nilsson 1989), i.e. they became important parts of community building and a powerful element in processes of social change.

However, the testimonies presented also show that lifestyle entrepreneurs are able to survive commercially partly because they can rely on each other and develop mechanisms of integration within the foreign community (e.g. Hub association but also Jamie Malloy’s JCJ bar) and also between the foreign and local community, and as described by Cunha et al. (2018):

It seems that the culture of promoting sharing, exchange and, in many cases, the sale of surpluses from their small production is relatively established in their own organization and organization of short marketing channels. The search for mutual assistance in agricultural work—and others—is a current practice. (p.72)

This is consistent with Ateljevic and Doorne (2000) and Klapper et al. (2018) conclusions that the foreign immigrant community is significantly reliant on their own resources and those of the group, making evident the importance of social capital for the success of their ventures.

But the presence of these immigrants with new habits and more purchasing power has created a demand that also impacts the economic activities promoted by local people, originating new businesses and making the existing ones more viable, as shown in Box 7.

#### Box 7 Impact of lifestyle immigrants in local businesses

“*In the center of Penamacor there is a gourmet grocery store that had to adapt to the arrival of outsiders. ‘Do you know what they buy most?’ asks Érica Bargão, who has occupied the space behind the counter since the store opened three years ago. ‘Dog food. Here, we give the animals the leftover food, but they prefer it that way. So we had to reinforce the orders. That, the tea, the bacon. And then we started introducing new products that were not used here, like oats and lentils’. (…) This store opened when the first foreigners started to arrive, Érica knows that it was this impetus that came from outside that is making the business profitable”.* (#9L *Diário de Notícias-14/10/2018*)

“*It is noticeable! In the morning, it’s almost double … it’s 50/50: 50 Portuguese, 50 English. And, at night, you can also notice (…) because they have no problems about spending money. (…) I think it will be our future, because the people get old, the new ones don’t stay and so, I think they will be our hope in Penamacor*”. (#11L, TVI24, 01-11-2018)

*“I don’t know how to speak… I only know how to speak Portuguese, but I have already transported them, that I am a taxi driver … they are more … hum … how can I say they are more ‘thing’ than the Portuguese…the Portuguese are always haggling over the price and they are not, what the taximeter mark is what they pay”.* (#12L, TVI24, 01-11-2018)

These new inhabitants in Penamacor county also generated a demand for other basic services, such as health, education and information technologies, which created a “critical mass” (Dinis 2006) that justify/make viable the creation/development of these infrastructures in the territory. For instance, in September 2017, the first English curriculum school in the country opened here. Being fundamental to the quality of life of families, the existence of those services became elements of attraction for more immigrants, particularly for the active and younger ones (see Box 8).

#### Box 8 Impact of lifestyle immigrants in local infrastructures

*“The health center not only gained more than one hundred of users but also strengthened valences: ‘increased maternal health, because some have already become pregnant, some women (…) and the family planning…it all increased’*”. (#4L, SIC Notícias, 25/03/2019)

*“At the health center, the head nurse Vítor Fernandes explains the impact of the arrival of children: ‘Until now, we were almost a palliative unit. We served almost exclusively old people who need continued care. And now we have children again, we can already do prevention campaigns, vaccinations, teach healthy habits. And this is what we really are for”.* (#5L, Diário de Notícias-14/10/2018)

*“The [former] day school of Our Lady of Incense [closed since the 90s for lack of students] gave way to the only school in English in the interior of Portugal [The international school of Penamacor.”(…) ‘There were 33 students now, from 4 and 12 years old and from more than 10 different countries: Israel Canada, America, England, Germany, Italy, France …’ “(#21 [teacher/director of the school], TVI24, 01-11-2018).” The school began two years ago with a lady call Zoe Burgess who had a home tuition school in the U.K and she moved to Portugal and wanted to continue that for her children. Other local foreign children joined her and she was very successful, and then needed a larger building and the municipality provided her this building (…) I imagine that in the next couple of years, with the people that I’m talking to and that are coming to the area, the school will probably reach 60 students in the (…) and then will increase after that*” (#21 Diário de Notícias-19/10/2018)

*“I have a Portuguese wife (…) she is from Oporto. We are both teachers and we were looking for the right school (we have two children) to put them and then we found this school that was here in Penamacor…”* (#8, Diário de Notícias-19/10/2018)

*“There are also three dozen foreign children attending Portuguese education, and this allows in some cases to open two classes for the same year, something that was not mentioned in Penamacor for decades”.* (Diário de Notícias-14/10/2018)

*“Today we have in most of our villages and in our parishes, a fiber optic network, that is, working here in the village of João Pires is perfectly the same as working in the center of the capital or there is not a climate of differentiation in terms of job opportunities. (#1L, SIC, 25/03/2019) “We are about to tender for the opening of a technological center. Basically, we will have rooms with internet with fiber, so that it is possible to work in this land with high speed internet”*. (#1L, Diário de Notícias-14/10/2018)

Furthermore, the presence of these global citizens in the territory also generates other mobility flows, attracting others—mainly family and friends—as tourists (for short periods) or as immigrants (for longer periods or for life) (see Box 9), generating a virtuous cycle, i.e. reinforcing the local dynamic of economic growth and social development. It should also be noted that in the study of Cunha (2018), from the first phase (January 2017) to the second phase of data collection (which includes questionnaires from April and May 2018), the answer “I was advised by friends” increased from 3% to 17% of respondents, showing a marked growth in the “word of mouth”.

#### Box 9 The generation of new influxes of people

*“I came here five years ago because I was told that there was really cheap land here. I bought a farm and started to tell some friends, especially English, that there were fantastic opportunities here. And some came along”. (#9, DN, 2018)*

*“11 years ago he bought a farm with about 1.3 hectares, near Aldeia de João Pires where he lives alone most of the time, except when he is visited by one of his 4 children and 3 grandchildren who live in England”. (#3,* Cunha 2018*:64)*

*“She bought a 2.2-hectare farm near the village of Pedrógão, rebuilt the house in ruins to the minimum conditions and lives there alone, except when she receives a visit from his son who lives in England and his friends. Recently, the son launched on social media a request for volunteering to help with the farm and rebuilding the house, and now he is preparing to receive, during next summer, groups of 4 volunteers out of a total of 14 who responded to the request”. (#5,* Cunha 2018: 64)

## Conclusions

The purpose of this study was to explore the nexus between tourism and migration and, through the lens of the lifestyle entrepreneurship, to assess the impact of the new patterns of mobility in rural and low density territories. The case of Penamacor, one of the most lagging regions in Portugal, represents a paradigmatic case of this new motilities, presenting a recent and overwhelming influx of young (and not so young) people to the territory. Thus, this case was instrumental to further understand *who these migrants* are and what their motivations are for mobility and to settle in the territory and also if *entrepreneurship is a possibility of income generation for these immigrants and for the creation of wealth and well-being in the community*. It was also intended to understand whether the presence of these new incomers in the territory *generates other mobility flows*, through the attraction of others, mainly, family and friends or acquaintances, as tourists, immigrants or travellers.

Results show that in spite of their lifestyle motivations, they are not all the same, showing differences in their demographic characteristics, specific motivations, occupations and their patterns of mobilities and spatial frames of action. They differ in their ages, including retired people and—mostly—young people, who came alone or with family. They also differ in their motivations with some of them mainly concerned with their (and their family) quality of life, not necessarily implying a radical detachment from the capitalist/market system, whilst others are clearly motivated by socio-ideological beliefs associated with the rejection of the mainstream neoliberal order, looking for alternative ways of living. Also, in spite of the fact that most practice agriculture, it seems that there are also differences in the weight that this activity represents in their income and working hours. In some cases, they abandoned their previous professional occupations to dedicate themselves to agriculture in full time or complementary to other local activities. However, in other cases, agriculture seems to be a hobby, in the sense that their professional occupation and what their source of income are, mainly, connected to the same (or similar) jobs as before migration, working globally and making extensive use of information technologies and telework. In general, these new inhabitants have in common the extended/international “life experiences” and often a history of international mobilities, making them “citizens of the world” and “travellers” (as defined by Ateljevic and Doorne 2000), with different perspectives and attitudes, from tourists and traditional immigrants, in regard to mobility. However, in addition to the above finer distinction concerning lifestyle motives and age (and thus in concerning their position in the market labour—active/inactive), they also differ in the patterns of permanence in the territory, with some presenting a stable permanence in the territory (some for life) and others presenting a circulation/shifting mode between one or several international locations. These differences can be aggregated in different types of lifestyle migration: *retirement migration*, *stable migration* and *nomad* or *peripatetic migration*. These typologies can be better understood in a multidimensional scheme, along four dimensions: (i) age/situation in the labour market (active vs retired); (ii) individual or family lifestyle vs social/ideological motives; (iii) frame of action (occupation); and (iv) duration of permanence in the territory. *Retired immigrants* are distinguished from the rest by the fact that they are no longer active in the labour market and, therefore, there is no need to balance work with quality of life. For the same reason, it makes no sense to talk about the spatial frame of their (professional) occupations. *Stable lifestyle immigrants* differentiate from nomad by their permanence in the territory, corresponding to a more stable pattern (possibly definitive) in opposition to the “circulation” mode of the “nomad”. Associated with the nomad’s circulation mode is their professional global/non-local work context.

For what concerns their impact on the community, results show that immigrants’ characteristics, namely, their global experience and perspectives, their (younger) age and higher qualifications than native population, can make them more aware of business opportunities and also agents of social and cultural change, acting both as commercial and communitarian entrepreneurs. Furthermore, the success of their ventures is strongly supported by their own (foreign) community, as both clients and partners. Besides that, their presence in territory increased the demand for local products and services and create a “critical mass” (Dinis 2006) needed for the viability of essential infrastructures as health and education. Finally, through their social networks—family, friends and acquaintances—other mobility flows to the territory are generated whether for long or short periods of time, reinforcing the local dynamic of economic growth and social development.

From the theoretical point of view, the study provides more in-depth understanding of emigrant lifestyles, showing that they do not constitute a homogeneous group and designing new differentiated profiles. It also confirms the relevant economic and social impact that these new inhabitants have on a territory that suffers from depopulation problems. The study further relates literature with different traditions, namely, exploring the tourism-migration nexus and associating it with the field of entrepreneurship, providing, at the same time, important clues for rural studies and the field of regional development. Finally, the study reinforces and extends to other sector besides tourism, the conclusion of Williams et al. (1989) about the blurring borders between consumption (lifestyle)-led and production (entrepreneurial)-led migration. From a practical point of view, the study advances knowledge about the characteristics, motivations, capacities and (potential) impacts of these new settlers, which is essential to design policies aimed at attracting new settlers, supporting their projects and leveraging their impact on the territory.

In spite of these contributions, this study had an eminently qualitative and exploratory nature, so, more than providing definitive answers, it allowed to further understand *why* these immigrants come and *how* they impact the territory. However, more studies of descriptive and quantitative nature are needed in order to provide more conclusive evidence concerning the profiles and the impact. For instance, future research should measure the relative weight of each type of immigrant and to quantify the impact of their presence and activities and, also, to assess if different migrant profiles have different impacts in the territory.

Furthermore, other questions can be deepened in further studies. For instance, as noted by Marchant and Mottiar (2011), “as life changes so do motivations and desires”; thus, along their life cycle, an individual can move from being one type of immigrant to another or from being one type of entrepreneur to another, for instance, if a “nomad” become “stable” or a “lifestyle” entrepreneur become commercial one. Why does it happen? What are the implications of such changes? How often does it happen? These are also questions to be answered in future research.

The present work focusses on a chapter of the migrations (hi)story in a low-density territory that will be succeeded by other chapters that are important to follow. For example, in Penamacor, the academic year 2019–2020, the international school did not open due to problems in the internal dynamics. What impact will the closure of the school have on the sustainability and growth of the immigration? If the migratory flow continues to increase significantly, how is it support the integration of these foreign immigrants preserving also the identity of the local community? Additionally, in 2020 we are facing the pandemic of COVID-19, which profoundly affected the mobility and the relationship with spaces. What will the future impact of this threat (or similar ones) in these territories be? Will containment of mobility have longer term effects, restraining the globalization process and consequently also the international migratory movements? Or, on the contrary, will it accentuate the counterurbanization movement, by people searching for greater health security in less densely populated areas? These are some of the challenges that these territories face and for which it is worth looking for answers.

## Sources of Empirical Data

### Personal Interview

Exploratory personal interview with Pedro Agapito, employee of the municipality of Penamacor and professor of Portuguese foreign community in Penamacor

### Studies/Reports

Report of the Foreign Community Monitoring Working Group presented at the Municipal Assembly of Penamacor February 2017

Cunha, Anselmo (2018). Neoruralidades em territórios de baixa densidade—o caso do concelho de Penamacor, Dissertação de mestrado em Antroplogia de Iberoamérica,/(Neoruralities in low-density territories—the case of the municipality of Penamacor, Master’s Dissertation in Anthropology of Iberoamerica), Universidad de Salamanca, June 2018

Vilas Boas, M. De Carvalho, C., Rodrigues, J. e Valente, A. (2015): Património Geológico de Penamacor: Inventário Geossítios e propostas para a sua valorização, *AÇAFA* On Line, n° 10 Associação de Estudos do Alto Tejo

### Statistical Data

Foreign and Borders Service (SEF), Number of registration certificates Issued for the municipality of Penamacor between November 2008 and February 2020

PORDATA, Data Sources: INE—Annual Estimates of the Resident Population; Data Sources: IGP—National Cartographic Series at 1:50,000 scale and Official Administrative Letter of Portugal—CAOP 2009.0. Last updated: 2020-02-07

PORDATA, Data Sources: INE—Annual Estimates of the Resident Population | SEF/MAI—População Estrangeira com Estatuto Legal de Residente, Last updated 2020-02-07

### Sites/Blogs

Municipality of Penamacor site: http://www.cm-penamacor.pt

Blog of Pedrogrão de S. pedro (Small village of Penamacor County): http://pedrogaosaopedro.blogspot.com/2017/03/penamacor-estrangeiros-estao-dar-vida.html

### News/Documentaries in Media

*In the Local Press*

Furtado, J (2017, 23 March). Estrangeiros estão a dar vida a penamacor [Foreigners are giving life to Penamacor]. *A Reconquista*, p. 22

*In the National Press*

Araújo, A. (2019, 3 March) Preço das casas na cidade empurra procura para aldeias do interior [Price of houses in the city pushes demand for rural villages] availabel in *Expresso,* Cadernos de Economia, https://expresso.pt/economia/2019-08-03-Preco-das-casas-na-cidade-empurra-procura-para-aldeias-do-interior

Faria, N. (2012, 16 July). A Aldeia de Martim está condenada [The Village of Martim is doomed]. *Público*, available in https://www.publico.pt/2012/07/16/jornal/a-aldeia-de-martim-esta-condenada-24898186

Rodrigues, R. (2018, 14 October) Os fugitivos do brexit estão a invadir o concelho mais envelhecido de Portugal [Brexit fugitives are invading Portugal’s oldest municipality], *Diário de Notícias*, available in https://www.dn.pt/edicao-do-dia/14-out-2018/os-fugitivos-do-brexit-estao-a-invadir-o-concelho-mais-envelhecido-de-portugal-9996873.html

*Documentaries (Video/TV)*

Ferreira, M. (journalist) & Ramos, A. (Coord) (2019, 27 March). Good Morning Penamacor. *SIC Notícias*, *Sic Notícias*, available in https://sicnoticias.pt/programas/reportagemsic/2019-03-27-Good-Morning-Penamacor [Duration:29m:46′)

Rodrigues, R. (2018, 19 October) Fugir do Brexit para Penamacor [Escape from Brexit to Penamacor], *Diário de Notícias*, https://www.dn.pt/galerias/videos/vida-e-futuro/fugir-do-brexit-para-penamacor-10027837.html (Duration:9m:11′)

(s/a., 2018, 2, November). Penamacor tem a maior taxa de residentes estrangeiros do interior [Penamacor has the highest rate of foreign residents in the interior], *TVI 24*, available in https://tvi24.iol.pt/videos/sociedade/penamacor-tem-a-maior-taxa-de-residentes-estrangeiros-do-interior/5bdca6a10cf2223b6a7ae10d (Duration: 4m:05′)

All together, these sources allowed access to statements of 27 foreign immigrants, corresponding to 21 households (Table 4) and 12 local inhabitants (Table 5), that constituted key informants for the empirical study.

**Table 4:** Foreign community key informants

#	Name	Gender	Age	Nationality	Source
1a	Jacques^a^	Male	44	French	Cunha (2018: 62–69)
1b	Brigitte^a^	Female	41	French	Cunha (2018: 62–69)
2a	Sílvia^a^/Flora van Heteren	Female	69	Dutch	Cunha (2018: 62–69) *SIC Notícias-27/03/2019*
2b	Eric^a^/Dudley Campling	Male	69	English	Cunha (2018: 62–69) *SIC Notícias-27/03/2019*
3	Ian^a^	Male	57	English	Cunha (2018: 62–69)
4a	Loorena^a^	Female	34	Iranian/English	Cunha (2018: 62–69)
4b	Chris^a^	Male	32	English	Cunha (2018: 62–69)
5	Sandy^a^/Sophia Mars	Female	51	English	Cunha (2018: 62–69) *Diário de Notícias-*14/10/2018 (press) *Diário de Notícias-*19/10/2018 (video)
6	Jack^a^	Male	48	Scottish	Cunha (2018: 62–69)
7a	Peter Siedle	Male	51	English	*Diário de Notícias*-14/10/2018 (press) *Diário de Notícias-*19/10/2018 (vídeo)
7b	Dawn Siedle	Female	51	English	*Diário de Notícias*-14/10/2018 (press) *Diário de Notícias-*19/10/2018 (vídeo)
8	Andrew Smith	Male	37	Australian	*Diário de Notícias*-14/10/2018 (press) *Diário de Notícias-*19/10/2018 (vídeo) *SIC Notícias-27/03/2019*
9	Jamie Molloy	Male	38/40	Irish	*Reconquista-27/03/2017* (press) *Diário de Notícias*-14/10/2018 (press) *Diário de Notícias-*19/10/2018 (vídeo) *TVI 24-02/11/2018* *SIC Notícias-27/03/2019*
10	Joshua Laurent	Male	36	English	*SIC Notícias-27/03/2019*
11	Tira Noren	Female	50	Swedish	*SIC Notícias-27/03/2019*
12	Helen Morgen	Female	57	Wales	*SIC Notícias-27/03/2019*
13	Peter Russeu	Male	47	English	*SIC Notícias-27/03/2019*
14	Shaun Kenny	Male	45	Wales	*SIC Notícias-27/03/2019*
15	Jesus Mathias	Male	33	American	*SIC Notícias-27/03/2019*
16a	Stephanie Bruns	Female	38	German	*SIC Notícias-27/03/2019*
16b	Janus Bruns	Male	44	German	*SIC Notícias-27/03/2019*
17	Carly Bennet	Female	43	English	*SIC Notícias-27/03/2019*
18a	Noélia Rodrigues	Female	32	Spanish	*SIC Notícias-27/03/2019*
18b	Richard Stonechild	Male	32	English	*SIC Notícias-27/03/2019*
19	Zahrah Nasir	Female	62	Pakistani/Scottish	*TVI 24-02/11/2018* *SIC Notícias-27/03/2019*
20	Figgs	Male	33	Zimbabwean	*SIC Notícias-27/03/2019*
21	Paul Large	Male	50	English	*Diário de Notícias*-14/10/2018 (press) *Diário de Notícias-*19/10/2018 (video) *SIC Notícias-27/03/2019*

^a^Fictitious names

**Table 5:** Local community key informants

#	Name	Identification	Sources
1L	Luis Bates	Mayor of Penamacor	*Diário de Notícias*-14/10/2018 (press) *Diário de Notícias-*19/10/2018 (vídeo) *TVI 24-02/11/2018* *SIC Notícias-27/03/2019*
2L	Pedro Agapito	Employee of the municipality of Penamacor and professor of Portuguese foreign community in Penamacor	Personal Interview (February 2020) *Reconquista-27/03/2017 (Press)* *SIC Notícias-27/03/2019*
3L	Anselmo Cunha	Sociologist from a Penamacor village (Aldeia do Bispo)	Master Dissertation (2018) *Reconquista-27/03/2017 (Press)* *SIC Notícias-27/03/2019*
4L	Elizabete Rato	Doctor at Penamacor Health Center	*SIC Notícias-27/03/2019*
5L	Vitor Fernandes	Head nurse at Penamacor Health Center	*Diário de Notícias*-14/10/2018 (Press)
6L	António Pinto	President of Pedrógrão de S. Pedro and Bemposta townships union (small villages of Penamacor county)	*SIC Notícias-27/03/2019*
7L	Gonçalo Martins	Kid from Penamacor (football player)	*SIC Notícias-27/03/2019*
8L	Cristina Canaveiro	Shopkeeper	*SIC Notícias-27/03/2019*
9L	Érica Bargão	Shopkeeper	*Diário de Notícias*-14/10/2018 (Press)
10L	Ilda Tavares	Local inhabitant	*SIC Notícias-27/03/2019*
11L	(Young man)	Bartender	*TVI 24-02/11/2018*
12L	(Old man)	Taxi driver	*TVI 24-02/11/2018*

## Footnotes

1 See Williams and Hall (2002) who explain how each of these factors contributed for increasingly mobile societies.

2 The distances from Penamacor to this places are all less than 50 km.

3 In English in the original.

4 PORDATA; Data Sources: INE|SEF/MAI – Foreign Population with Legal Resident Status (last update: 2019-07-30).

5 As Cunha (2018: 13) states, “it should be noted that these numbers are official, that is, they reflect only the expats who formally requested the residence certificate. In fact, the actual number of those residing in Penamacor may be higher. (…). The explanation for this difference may be that the majority of those who arrive with retired status and income do not apply for the SEF document, presumably because it is not rewarding for them for fear of losing the social support benefits in their country of origin”.

6 Study based on 38 questionnaires, applied to European foreign residents installed for more than 1 year in the municipality of Penamacor, including different nationalities and residing in rural areas (outside urban areas) and also a qualitative analysis, based on six interviews with expats (which involved nine people).

7 That represent more than 65% of individuals, and only 11% are older.

8 Touristic region in the south of Portugal where the largest English community in the country lives.

9 Organic farming is practiced by 71% of respondents, 45% exclusively and 26% together with conventional farming.

10 In a footnote.

11 In fact, the number that expressed that desire is exactly the same of those that already decided to stay for living in Penamacor.

12 Employee of the municipality of Penamacor and professor of Portuguese foreign community in Penamacor, in a personal interview.
